# Supplementary material for: Worldwide Research Trends on Milk Containing Only A2 β-Casein: A Bibliometric Study
Source: Animals (Basel). 2022 Jul 27;12(15):1909. doi: 10.3390/ani12151909 (PMC9367265; doi:10.3390/ani12151909)
Supplement: Supplementary file 1 [file animals-12-01909-s001.zip › animals-1762921-SI.pdf]

animals

Article

Worldwide Research Trends on Milk Containing only A2 β-casein: A Bibliometric Study

Lucía Jiménez-Montenegro [1], Leopoldo Alfonso [1], José A. Mendizabal and Olaia Urrutia [1,*]

[1] IS-FOOD, School of Agricultural Engineering and Biosciences, Public University of Navarre (UPNA), Campus de Arrosadia, 31006 Pamplona, Spain; lucia.jimenez@unavarra.es (L. J.-M.); leo.alfonso@unavarra.es (L. A.), jamendi@unavarra.es (J.A.M); olaia.urrutia@unavarra.es (O.U.).

* Correspondence: olaia.urrutia@unavarra.es

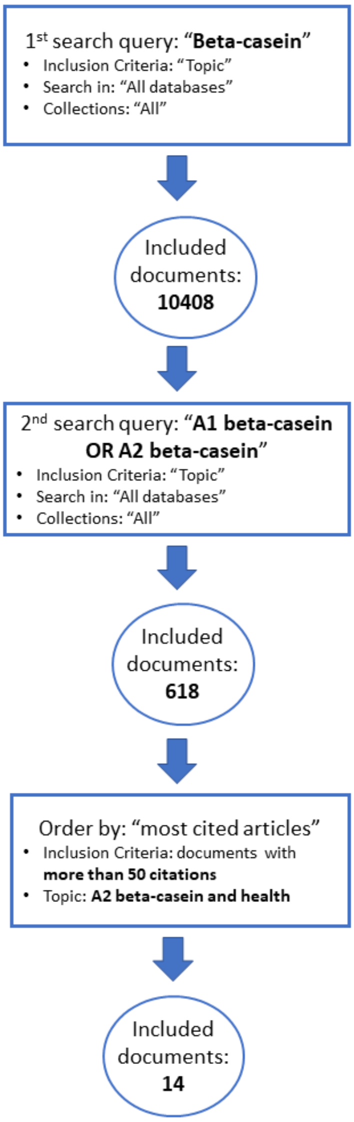

Figure S1. Methodological flowchart accomplished in this scientometric analysis.

Table S1. Top 10 most cited articles on A2 β-casein research field.

Number of cites	Title	Year	Affiliation	Funding agency	Document type	Reference
258	*Isolation and structural analysis of antihypertensive peptides that exist naturally in Gouda cheese*	2000	Univ. Tohoku (Japan)	-	Article	[1]
230	*Anti-inflammatory mechanisms of bioactive milk proteins in the intestine of newborns*	2013	Univ. Copenhagen (Denmark)	Strategic Research Councils	Review	[2]
214	*Substrate-specificity of cyclic AMP-dependent protein kinase*	1975	Univ. California (USA)	-	Article	[3]
200	*Effects of milk protein genetic-variants on milk-yield and composition*	1984	John Curtin School of Medical Research (Australia's national medical research)	-	Article	[4]
200	*Differential staining of phosphoproteins on polyacrylamide gels with a cationic carbocyanine dye*	1973	National Cancer Institute, NCI (Maryland, USA)		Article	[5]
177	*Polymorphism of bovine beta-casein and its potential effect on human health*	2007	Univ. Warmia (Poland)	-	Review	[6]
165	*Association of genetic-variants of casein and milk serum-proteins with milk, fat, and protein-production by dairy-cattle*	1984	McGill Univ. (USA)	-	Article	[7]
159	*Effects of milk protein variants on the protein composition of bovine milk*	2009	Univ. Wageningen (Netherlands)		Article	[8]
151	*STAT5A-deficient mice demonstrate a defect in granulocyte-macrophage colony-stimulating factor-induced proliferation and gene expression*	1997	FDA (USA); National Institute of Diabetes and Digestive and Kidney Diseases, NIDDK (USA)	-	Article	[9]
136	*Type I (insulin-dependent) diabetes mellitus and cow milk: casein variant consumption*	1999	Univ. Auckland and New Zealand Dairy Research Institute (New Zealand) and Univ. Düsseldorf (Germany)	-	Article	[10]

REFERENCES

1. Saito, T.; Nakamura, T.; Kitazawa, H.; Kawai, Y.; Itoh, T. Isolation and Structural Analysis of Antihypertensive Peptides That Exist Naturally in Gouda Cheese. *Journal of Dairy Science* **2000**, *83*, 1434–1440, doi:10.3168/jds.S0022-0302(00)75013-2.
2. Chatterton, D.E.W.; Nguyen, D.N.; Bering, S.B.; Sangild, P.T. Anti-Inflammatory Mechanisms of Bioactive Milk Proteins in the Intestine of Newborns. *International Journal of Biochemistry and Cell Biology* **2013**, *45*, 1730–1747, doi:10.1016/j.biocel.2013.04.028.
3. Kemp, B.E.; Bylund, D.B.; Huang, T.-S.; Krebs, E.G. Substrate Specificity of the Cyclic AMP-Dependent Protein Kinase (Caseins/Protein Sequence). **1975**, *72*, 3448–3452.

4. Mckenzie, H.A.; Brucec Graham, E.R.; Ponzoni, R.W. Effects of Milk Protein Genetic Variants on Milk Yield and Composition. *Journal of Dairy Research* **1984**, *51*, 531–546, doi:10.1017/S0022029900032854.
5. Green, M.R.; Pastewka, J. v.; Peacock, A.C. Differential Staining of Phosphoproteins on Polyacrylamide Gels with a Cationic Carbocyanine Dye. *Analytical Biochemistry* **1973**, *56*, 43–51, doi:10.1016/0003-2697(73)90167-X.
6. Kaminski, S.; Cieslinska, A.; Elzbieta, K. Polymorphism of Bovine Beta-Casein and Its Potential Effect on Human Health. *J. Appl. Genet.* **2007**, *48*, 189–198.
7. Ng-Kwai-Hang, K.F.; Hayes, J.F.; Moxley, J.E.; Monardes, H.G. Association of Genetic Variants of Casein and Milk Serum Proteins with Milk, Fat, and Protein Production by Dairy Cattle. *Journal of Dairy Science* **1984**, *67*, 835–840, doi:10.3168/JDS.S0022-0302(84)81374-0.
8. Heck, J.M.L.; Schennink, A.; van Valenberg, H.J.F.; Bovenhuis, H.; Visker, M.H.P.W.; van Arendonk, J.A.M.; van Hooijdonk, A.C.M. Effects of Milk Protein Variants on the Protein Composition of Bovine Milk. *Journal of Dairy Science* **2009**, *92*, 1192–1202, doi:10.3168/JDS.2008-1208.
9. Feldman, G.M.; Rosenthal, L.A.; Liu, X.; Hayes, M.P.; Wynshaw-Boris, A.; Leonard, W.J.; Hennighausen, L.; Finbloom, D.S. STAT5A-Deficient Mice Demonstrate a Defect in Granulocyte-Macrophage-Stimulating Factor – Induced Proliferation and Gene Expression. *Blood* **1997**, *90*, pp 1768-1776.
10. Elliott, R.B.; Harris, D.P.; Hill, J.P.; Bibby, N.J.; Wasmuth, H.E. Type I (Insulin-Dependent) Diabetes Mellitus and Cow Milk: Casein Variant Consumption. *Diabetologia* **1999**, *42*, 292–296, doi:10.1007/s001250051153.
